# Prevention and Therapeutic Effects and Mechanisms of Tanshinone IIA Sodium Sulfonate on Acute Liver Injury Mice Model

**DOI:** 10.1155/2016/4097398

**Published:** 2016-05-04

**Authors:** Lunjie Lu, Jun Zhou, Jingying Zhang, Jun Che, Yang Jiao, Yusong Zhang

**Affiliations:** ^1^Department of Oncology, The Second Affiliated Hospital of Soochow University, Suzhou 215004, China; ^2^Department of Radiation Genetics, School of Radiation Medicine and Protection, Soochow University School of Medicine, Suzhou 215123, China; ^3^Department of Emergency, Suzhou Kowloon Hospital, Shanghai Jiao Tong University School of Medicine, Suzhou 215000, China; ^4^Department of Radiation Oncology, The Affiliated Hospital of Jiangnan University, Wuxi 214062, China

## Abstract

Tanshinone IIA sodium sulfonate (TSS) is a water-soluble derivative of tanshinone IIA, which is the main pharmacologically active component of Salvia miltiorrhiza. This study aimed to verify the preventive and therapeutic effects of TSS and its combined therapeutic effects with magnesium isoglycyrrhizinate (MI) in D-galactosamine- (D-Gal-) induced acute liver injury (ALI) in mice. The potential regulatory mechanisms of TSS on ALI were also examined. Our results may provide a basis for the development of novel therapeutics for ALI.

## 1. Introduction

Acute liver injury (ALI) is pathologically characterized by rapid-onset hepatocellular necrosis, apoptosis, and liver inflammation in patients without preexisting liver disease [1]. The most clinically serious case of ALI is acute liver failure [2], which exhibits typical clinical manifestations, such as coagulopathy, hepatic encephalopathy, and multiorgan failure within a short time and with an unpredictable high mortality rate [3, 4]. The considerably high incidence of ALI makes it a serious public concern, and its pathogenesis seems to be versatile. The putative causes of ALI include hepatitis virus infection, alcohol intoxication, ischemia, and extensively used drugs such as chemotherapy agents, antipyretic analgesics, immune suppressants, antidiabetic drugs, antibacterial, antifungal, and antiviral agents, and diet pills [1, 5–7]. Therefore, preventive measures and cause-specific supportive therapy for ALI should be developed to optimize liver regeneration and to reduce mortality.

Tanshinone IIA sodium sulfonate (TSS) is a water-soluble derivative of tanshinone IIA (TSN), which is the most abundant and pharmacologically active diterpenoid quinone extracted from the dried root of* Salvia miltiorrhiza* Bunge (Danshen) [8]. As a commonly used traditional Chinese medicine, Danshen has been extensively investigated because of its multiple therapeutic potential against cardiovascular diseases, inflammation, diabetes, and metabolic syndrome; this potential has been supported by published papers, approved patents, and clinical trials in China and in the US [8–11]. TSN is an effective model of “one-drug-multi-target-multi-disease,” which provides a molecular basis for clinical applications to treat various kinds of diseases. TSN can also be combined with other drugs via different mechanisms to reduce side effects and to improve the pharmacodynamic and pharmacokinetic properties of agents [8]. However, in context of ALI pathogenesis, whether and how TSS could intervene in the whole process were not fully understood.

In this study, the preventive and therapeutic effects of TSS and its combined therapeutic effects with magnesium isoglycyrrhizinate (MI) in D-galactosamine- (D-Gal-) induced ALI in mice were studied. The potential regulatory mechanisms of TSS on ALI were also examined. Our study may provide a basis for the development of novel therapeutics for ALI.

## 2. Materials and Methods

### 2.1. Materials and Chemical

TSS was purchased from No. 1 Biochemical & Pharmaceutical Co., Ltd. (Shanghai, China). D-Gal was supplied by Run Cheng Biological Technology Co., Ltd. (Shanghai, China). MI was produced by Zheng Da Tian Qing Pharmaceutical Group Co., Ltd. (Lianyungang, Jiangsu, China). Mouse high-mobility group protein B1 (HMGB1) ELISA kit and mouse TGF-*β*1 Platinum ELISA kit were purchased from Shanghai WesTang Bio-Tech Co., Ltd. (Shanghai, China), and eBioscience Inc. (San Diego, CA, US), respectively.

### 2.2. Animals

Fifty 7-week-old female C57BL/6J mice were purchased from SLAC Experimental Animal Limited Liability Company (Shanghai, China). The mice were housed in cages (*n* = 5 in each cage) in a specific pathogen-free (SPF) environment. All items used to feed the mice were stringently sterilized. The mice were taken care of in the Laboratory Animal Center of Soochow University. Animal studies were approved by the Committee of Animal Care and Use for Research and Education at Soochow University (Suzhou, Jiangsu, China).

### 2.3. Induction of Acute Liver Injury and Treatment

D-Gal (450 mg·kg^−1^) was injected intraperitoneally for ALI model construction. All mice were randomly divided into 5 groups, ten mice per group. To investigate the preventive effects of TSS, the mice were pretreated with 100 mg·kg^−1^ TSS 2 h before D-Gal exposure. To identify the therapeutic effects of TSS, 2 h following D-Gal exposure, the mice were injected with 100 mg·kg^−1^ TSS. To evaluate the combined therapeutic effect, 100 mg·kg^−1^ TSS and 45 mg·kg^−1^ MI were applied 2 h after D-Gal injection. In the untreated control group, 10 mice were randomly chosen for 200 *μ*L physiological saline i.p. injection.

At certain time points, the mice were euthanized. The peripheral blood was collected, and the serum was detected. The livers were excised for further analysis.

### 2.4. Serum Protein and Enzyme Detection

Blood was centrifuged at 3000 rpm·min^−1^ for 10 min, and 200 *µ*L of the upper serum layer was collected to determine serum ALT and AST by using an Abbott automatic blood biochemical analyzer (Abbott Diagnostics, IL, USA). Serum HMGB1 and TGF-*β*1 levels were evaluated by using specific enzyme-linked immunosorbent assay kits in accordance with the manufacturer's instructions [12, 13]. In brief, 100 *μ*L serum and standard samples were added to duplicate wells and were incubated for 2 hours at room temperature. The samples were then aspirated, and all the wells were washed with 300 *μ*L washing buffer 3 times. For each well, 100 *μ*L of the secondary antibody in dilution buffer was applied and incubated for 1 hour at room temperature. Repeat the aspiration and washing procedure described above. Then, 200 *μ*L substrate solution was added to each well and incubated for 15 min at room temperature. The colorimetric reaction was stopped by adding 50 *μ*L of stop solution per well. The absorbance at 450 nm was determined using a microplate reader. The concentrations of HMGB1 and TGF-*β*1 were determined according to the standard curve constructed using the mean absorbance for each standard on the *y*-axis against the concentration on the *x*-axis.

### 2.5. Histology and Immunohistochemistry

The liver tissues were fixed with 4% phosphate-buffered paraformaldehyde and were embedded in paraffin. The tissues were sectioned in 5 *μ*m slices, stained with hematoxylin and eosin, and examined under light microscopy [14]. For immunohistochemistry staining, ten tissue sections per group were incubated in 0.25 mM EDTA at 95°C followed by additional 20 min incubation at room temperature for antigen retrieval. Nonspecific binding sites were blocked by 10% normal goat serum. IHC staining was performed with a primary rabbit anti-mouse cyclin D1 (EPR2241) monoclonal antibody (Abcam, MA, USA, diluted 1 : 1000) in accordance with standard immunohistochemistry procedure via diaminobenzidine visualization by using a DAKO EnVision TM+/HRP rabbit/mouse kit (Dako, Denmark A/S), as previously described [14]. The expression was quantified on the basis of digitally acquired images (10 fields/slide) by using Image-Pro Plus® Imaging (Molecular Devices, Inc., Sunnyvale, CA).

### 2.6. Western Blot Assay

Western blot assay was performed in accordance with previously described methods [14]. In brief, 1 mL of radiation-immune precipitate was used as lysis buffer supplemented with an inhibitor of protease cocktail (Roche Life Science, IN, US). Protein was extracted from the liver tissue and quantified through a BCA protein assay (Beyotime, Haimen, Jiangsu, China). Total protein (50 *µ*L) was separated from each sample through polyacrylamide gel electrophoresis (SDS-PAGE) followed by polyvinylidene fluoride membrane (PVDF) transmembrane reaction. The following primary antibodies were utilized: rabbit anti-cyclin D1 (EPR2241) monoclonal antibody (Abcam, MA, USA, 1 : 1000), rabbit anti-Akt (C67E7) monoclonal antibody (Cell Signaling Technology, MA, USA, 1 : 1000), rabbit anti-ERK (48H2) monoclonal antibody (Cell Signaling Technology, MA, USA, 1 : 1000), mouse anti-TGF beta 1 (2Ar2) monoclonal antibody (Abcam, MA, USA, 1 : 500), rabbit anti-Smad3 (EP568Y) monoclonal antibody (Abcam, MA, USA, 1 : 1000), rabbit anti-HMGB1 (EPR3507) monoclonal antibody (Abcam, MA, USA, 1 : 1000), rabbit anti-NF-*κ*B (48H2) monoclonal antibody (Cell Signaling Technology, MA, USA, 1 : 1000), rabbit anti-phospho-NF-*κ*B (Cell Signaling Technology, MA, USA, 1 : 1000) antibody, rabbit anti-phospho-I*κ*B-*α* (14D4) antibody (Cell Signaling Technology, MA, USA, 1 : 1000), and anti-GAPDH (Beyotime Biotechnology, Haimen, China, 1 : 1000) antibody. The membranes were then incubated with the corresponding secondary antibodies and subjected to chemiluminescent reagent reaction and X-film exposure.

### 2.7. Statistics

Data were expressed as means ± standard deviation and analyzed with SPSS version 17.0 (IBM, Chicago, USA). Statistical significance was evaluated through one-way ANOVA. *P* < 0.05 was considered statistically significant.

## 3. Results

### 3.1. TSS Pretreatment and TSS-MI Treatment Alleviated D-Gal-Induced Liver Injury

**Figure 1:**
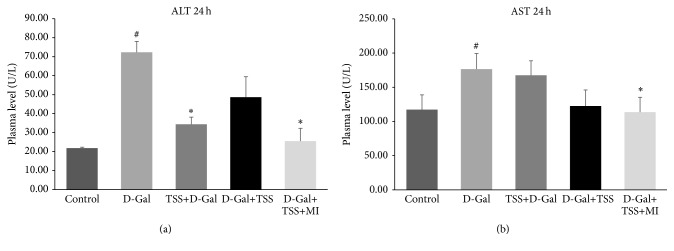
Effects of TSS pretreatment, single-drug treatment, and combined treatment with MI on mice with D-Gal-induced ALI. Mice were pretreated with 100 mg·kg^−1^ TSS 2 h before D-Gal (450 mg·kg^−1^) exposure; otherwise, they were treated with 100 mg·kg^−1^ TSS or 100 mg·kg^−1^ TSS together with 45 mg·kg^−1^ MI 2 h after D-Gal exposure. At 24 h after the treatments were administered, blood samples were harvested, and (a) alanine aminotransferase (ALT) and (b) aspartate aminotransferase (AST) levels were determined. Data were expressed as mean ± SD,* n* = 5. ^#^ *P* < 0.05 compared with the control group using Student's *t*-test. ^*∗*^ *P* < 0.05 compared with the D-Gal group using one-way ANOVA followed by Dunnett's analysis.

The aim was to determine the effect of TSS pretreatment and treatment on the liver function of the experimental mice with ALI. Five mice were randomly selected from each group, and the serum ALT and AST were evaluated 24 h after the mice were exposed to D-Gal. In Figure 1, ALT and AST levels were higher in the D-Gal-induced ALI group than in the control group (*P* < 0.05). The ALT levels in the TSS pretreatment group and the TSS-MI combination treatment group were significantly lower than those of the D-Gal-induced ALI group (*P* < 0.05). The TSS pretreatment and treatment could decrease the AST level, but the combined TSS and MI treatment significantly decreased the serum AST level (*P* < 0.05).

### 3.2. TSS Pretreatment and Treatment Relieved the Histopathological Variations of ALI Liver Tissues

**Figure 2:**
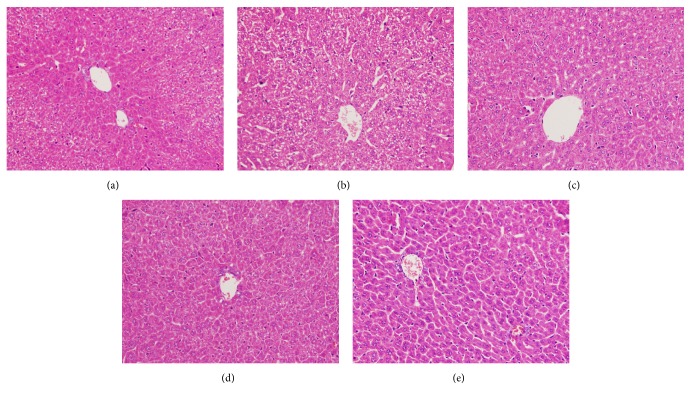
TSS improves liver histological alterations in mice with D-Gal-induced ALI. Morphological changes in liver tissues were observed through liver section hematoxylin-eosin staining. (a) Mice treated with physiological saline; (b) mice exposed to D-Gal (450 mg·kg^−1^); (c) mice pretreated with TSS (100 mg·kg^−1^) before D-Gal exposure; (d) mice treated with TSS (100 mg·kg^−1^) alone after D-Gal exposure; (e) mice treated with TSS (100 mg·kg^−1^) and MI (45 mg·kg^−1^) after D-Gal exposure. Representative liver sections of each group are shown (original magnification: 400x).

The liver tissues of each group were fixed in 10% neutral buffered formalin, embedded in paraffin, sliced into 4 *µ*m sections, and stained with hematoxylin and eosin. In the D-Gal-exposed group, hepatocyte edema, karyopyknosis, dissolved nucleus, ruined hepatic cords, and infiltrated inflammatory cells were observed. These findings indicated apoptosis and necrosis (Figure 2(b)). The liver injuries in the TSS treatment group comprised almost the same structure as the control group with normal hepatic cells, central veins, and lobules (Figures 2(a) and 2(d)). By contrast, TSS pretreatment and TSS-MI combination groups contained less hepatocytes necrosis than the D-Gal-exposed group did; the regenerated hepatocytes were observed in all of the three TSS treatment groups (Figures 2(c)–2(e)).

### 3.3. Enhancement of the Liver Regeneration by TSS Pretreatment and Treatment

**Figure 3:**
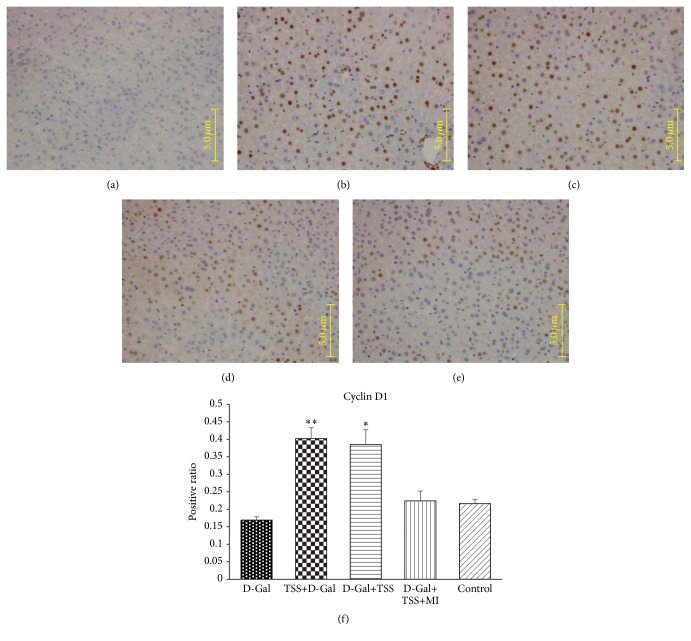
Effects of TSS pretreatment or treatment on cyclin D1 expression in D-Gal-exposed mouse liver. Cyclin D1 expression was detected through immunohistochemical staining. The dark-brown nucleus indicated cyclin D1-positive cells. (a) Mice treated with physiological saline; (b) mice exposed to D-Gal (450 mg·kg^−1^); (c) mice pretreated with TSS (100 mg·kg^−1^) before D-Gal exposure; (d) mice treated with TSS (100 mg·kg^−1^) alone after D-Gal exposure; (e) mice treated with TSS (100 mg·kg^−1^) and MI (45 mg·kg^−1^) after D-Gal exposure. Representative liver sections of each group are shown (original magnification: 400x). ^*∗∗*^ *P* < 0.01 compared with the D-Gal group; ^*∗*^ *P* < 0.05 compared with the D-Gal group, using one-way ANOVA followed by Dunnett's analysis.

Cyclin D1, which has been reported to promote liver cell regeneration, was subjected to immunohistochemical staining to evaluate the effects of TSS pretreatment or treatment on the liver regeneration of the D-Gal-induced ALI mice. At 24 h, cyclin D1 expression in all of the three treatment groups was higher than that of the D-Gal-exposed group (Figure 3). Cyclin D1 expression in TSS pretreatment and treatment groups was separately increased 2.6- and 2.3-fold compared with that of the D-Gal group (*P* < 0.01, by Student's *t*-test). The combined treatment of TSS and MI did not significantly increase cyclin D1 expression.

### 3.4. TSS Affected the Serum HMGB1 and TGF-*β*1 of Mice with ALI

**Figure 4:**
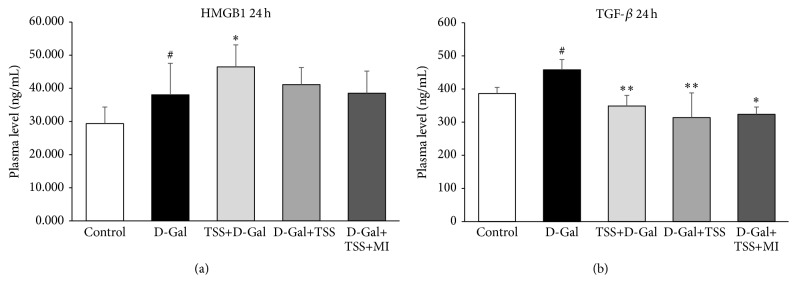
Effects of TSS pretreatment or treatment on serum HMGB1 and TGF-*β* levels in D-Gal-exposed mice. Serum HMGB1 (a) and TGF-*β* (b) levels were determined using ELISA detection kits. Control: mice treated with physiological saline; D-Gal: mice exposed to D-Gal; TSS+D-Gal: mice pretreated with TSS before D-Gal exposure; D-Gal+TSS: mice treated with TSS; D-Gal+TSS+MI: mice treated with TSS and MI after D-Gal exposure. Data were expressed as mean ± SD,* n* = 6. ^*∗∗*^ *P* < 0.01 compared with the D-Gal group; ^*∗*^ *P* < 0.05 compared with the D-Gal group, using one-way ANOVA followed by Dunnett's analysis; ^#^ *P* < 0.05 compared with the control group using Student's *t*-test.

The serum TGF-*β*1 and HMGB1 were evaluated 24 h after D-Gal induction and drug treatment were facilitated. Compared with the control group, D-Gal exposure significantly increased the serum HMGB1 and TGF-*β*1 levels (*P* < 0.05; Figure 4). The serum TGF-*β*1 levels in TSS pretreatment (*P* < 0.01), TSS treatment (*P* < 0.01), and TSS-MI combined treatment groups (*P* < 0.05) were lower than that in the D-Gal-exposed group. In contrast to D-Gal exposure, the other treatments could not significantly suppress the serum HMGB1 level. By contrast, the HMGB1 level in the TSS-pretreated group was higher than that of the D-Gal-exposed group at this time point (*P* < 0.05).

### 3.5. TSS Conducted the Preventative and Therapeutic Effects on ALI via the TGF-*β* and NF-*κ*B Signaling Pathways

**Figure 5:**
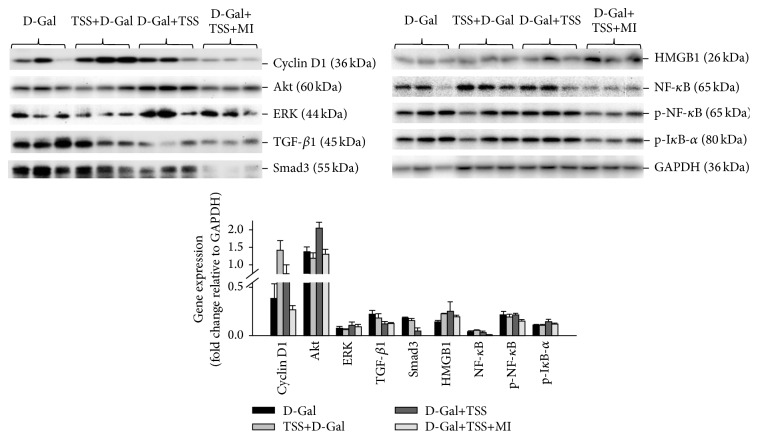
Effects of TSS pretreatment or treatment on the protein expression in the livers of the mice with ALI. Liver samples were harvested 24 h after D-Gal exposure and drug treatment. Hepatic protein expression was determined through Western blot. D-Gal: mice exposed to D-Gal; TSS+D-Gal: mice pretreated with TSS before D-Gal exposure; D-Gal+TSS: mice treated with TSS; D-Gal+TSS+MI: mice treated with TSS and MI after exposure to D-Gal. Expression level was shown as fold changes in band density.

The protein levels of cyclin D1 and TGF-*β* and NF-*κ*B signaling effectors were detected in the liver tissues through Western blot assay to investigate the possible mechanisms related to the influences of TSS on mice with ALI. Compared with D-Gal exposure, the TSS pretreatment highly increased cyclin D1 expression, moderately increased cellular HMGB1 and NF-*κ*B contents, and suppressed Akt, TGF-*β*1, and Smad3 expression after 24 h (Figure 5). The TSS treatment alone enhanced the expression of Akt, ERK, cyclin D1, HMGB1, and p-I*κ*B-*α*. By contrast, TGF-*β*1 and Smad3 expression decreased. The combined application of TSS and MI did not induce cyclin D1 expression in the tissues with ALI. However, this treatment impaired TGF-*β*1 expression and NF-*κ*B phosphorylation and decreased the cellular Smad3 and NF-*κ*B protein levels.

## 4. Discussion

Gal is a hepatotoxic 6-carbon amino sugar galactose derivative, which can cause hepatocyte necrosis and apoptosis. The short-term administration of Gal to small experimental animals is widely accepted as an appropriate model to investigate the pathogenesis and treatment of acute liver damage and acute liver failure [15–17]. In this study, the mice were treated with D-Gal for 24 h, and a mouse model of ALI was successfully constructed. The constructed model was confirmed through histological presentation and serum ALT/AST evaluation.

Previous studies demonstrated the protective and therapeutic effects of TSN against cardiovascular diseases, diabetes, neurodegenerative diseases, and various cancer types [8]. Moreover, the combined application of TSN with other drugs can potentiate the pharmacological effects via different mechanisms but can reduce the side effects of both agents by decreasing their dosage [8]. However, the clinical application of TSN has been limited because of its poor water solubility, oral bioavailability, and intestinal absorption [8].

Our study demonstrated the preventive, single-drug treatment and combined therapeutic effects of TSS, the water-soluble derivative of TSN, on D-Gal-induced ALI mice. Our results revealed that TSS elicited hepatoprotective effects. The TSS pretreatment administered before D-Gal exposure could significantly decrease the serum ALT level and relieve the damage to D-Gal-induced hepatocytes. The TSS single-drug treatment alleviated the D-Gal-induced increase in serum ALT and AST level, maintained the integrity of the liver tissues, and moderately increased cyclin D1 expression in the ALI liver tissues. These findings indicated hepatocyte regeneration. The combined treatment of TSS and MI, a clinically used hepatoprotectant, induced the most effective suppression of the increased serum ALT and AST levels, although histological evidence indicated that this phenomenon was not better than that observed in the TSS treatment alone. These results indicated that TSS potentiated the preventive and therapeutic effects against ALI at least at a preclinical level.

Similar to other traditional Chinese medicines, TSN elicits multitargeted effects [8, 18]. TSN has several potential molecular targets, including growth factors and receptors (e.g., TGF-*β*, AT1R, ER, AR, and VEGF), transcription factors (e.g., NF-*κ*B, Smad3, PPAR-*γ*, Nrf-2, and STAT-1), enzymes (e.g., AMPK, p38, ERK, PI3K/Akt, JNK, and MMPs), cytokines and chemokines (e.g., IL-1b, IL-6, and TNF-*α*), pro- and antiapoptotic proteins, ion channels and water channels, and certain microRNAs [8]. However, the related mechanisms and direct cellular and molecular targets of TSN remain unclear. TGF-*β*1 is a multifunctional growth factor that can bind to specific transmembrane receptors and initiate the phosphorylation of receptor-associated Smad2/3 proteins [19]. By recruiting Smad4, the activated receptors-proteins complex can regulate the transcription of target genes and perform various biological functions, such as proliferation suppression, immunosuppression, collagen synthesis promotion, and differentiation induction [19–22]. In this study, TSS pretreatment and single-drug treatment effectively suppressed the expression of TGF-*β*1 and Smad3. The combined treatment of TSS and MI almost inhibited the Smad3 expression without further suppressing the TGF-*β*1 expression. The results suggested that the preventive and therapeutic effects of TSS applied either alone or in combination with other chemicals were likely implicated in the regulation of the TGF-*β*/Smad pathway.

As a pivotal transcription factor for inflammatory mediators, the activated NF-*κ*B may exacerbate liver injury [23–25]. In resting cells, NF-*κ*B is inactivated by I*κ*B proteins but is activated via phosphorylation. When activated, NF-*κ*B is released from I*κ*B, translocated to the nucleus, and bound to the promoter regions of target DNA to activate gene transcription [24, 25]. In our study, TSS treatment inhibited the NF-*κ*B expression but increased the I*κ*B-*α* phosphorylation. The combination of TSS and MI suppressed the expression and phosphorylation of NF-*κ*B in liver tissues. The NF-*κ*B signaling in inflammation can also be activated by the interaction of HMGB1 and toll-like receptor [26, 27]. HMGB1 is an important chromatin protein and late inflammation mediator, which plays an extracellular role in cellular activation and proinflammatory responses [26]. Previous studies indicated that TSS can suppress HMGB1-associated activation of the NF-*κ*B pathway [27]. In our study, the HMGB1 expression was not suppressed by TSS 24 h after D-Gal and TSS treatment was administered. Nevertheless, our findings demonstrated that the NF-*κ*B signaling could be a major target of TSS in ALI.

TSS pretreatment and single-drug treatment may accelerate hepatocyte regeneration after D-Gal-induced ALI. The decreased Akt in the TSS pretreatment group, increased ERK expression in the TSS treatment group, and the increased cyclin D1 expression in both groups indicated that the same effect may be obtained via different mechanisms. Akt, also known as protein kinase B, is a major kinase in many cellular processes, such as glucose uptake, cell cycle progression, and apoptosis; as such, this molecule has been regarded as an important therapeutic target for the treatment of human diseases [28, 29]. As a major mediator of cell survival, Akt may directly inhibit proapoptotic proteins or signals generated by transcription factors [29]. Akt can also regulate NF-*κ*B signaling by phosphorylating IKK*α* and Tpl2 [29]. Our data revealed that the TSS pretreatment probably promoted hepatocyte regeneration by targeting Akt, which may simultaneously form a cross talk with NF-*κ*B signaling.

Extracellular signal-regulated kinase is a conventional mitogen-activated protein kinase involved in various fundamental cellular processes, such as proliferation, differentiation, motility, stress response, apoptosis, and survival [30, 31]. The activation of ERK promotes cell proliferation via EGFR ligands; thus, ERK has been extensively investigated as a target for cancer therapy. In our study, TSS treatment alone promoted the expression of ERK, and this phenomenon partly explained the difference between its therapeutic and inductive effects on Akt expression.

In summary, our findings suggested that TSS can prevent D-Gal-induced ALI and effectively cure ALI along or in combination with other hepatoprotectants. The multifunctions of TSS in ALI may be achieved through different mechanisms. TSS prevented D-Gal-induced ALI by targeting Akt. The therapeutic effects of TSS were mainly manifested as the inhibition of the TGF-*β*/Smad signaling cascade and the NF-*κ*B pathway. The combined treatment of TSS and MI could specifically target key transcription factors, such as Smad3 and NF-*κ*B. Our results indicated that TSS may be a potential choice for the prevention and treatment of ALI.
